# Satellite cells maintain regenerative capacity but fail to repair disease-associated muscle damage in mice with Pompe disease

**DOI:** 10.1186/s40478-018-0620-3

**Published:** 2018-11-07

**Authors:** Gerben J. Schaaf, Tom J. M. van Gestel, Stijn L. M. in ‘t Groen, Bart de Jong, Björn Boomaars, Antonietta Tarallo, Monica Cardone, Giancarlo Parenti, Ans T. van der Ploeg, W. W. M. Pim Pijnappel

**Affiliations:** 1 Department of Clinical Genetics, Erasmus MC, University Medical Center, Rotterdam, the Netherlands; 2 Department of Pediatrics, Erasmus MC, University Medical Center, Rotterdam, the Netherlands; 3 Center for Lysosomal and Metabolic Diseases, Erasmus MC, University Medical Center, Rotterdam, the Netherlands; 4 Department of Translational Medical Sciences, Federico II University, Naples, Italy; 5 Telethon Institute of Genetics and Medicine, Pozzuoli, Italy; 6 Present address: Department of Genetics, St Jude Children Research Hospital, Memphis, TN USA

**Keywords:** Satellite cells, Muscle regeneration, Pompe disease, Lysosomal storage disease, Glycogenosis type II

## Abstract

Pompe disease is a metabolic myopathy that is caused by glycogen accumulation as a result of deficiency of the lysosomal enzyme acid alpha glucosidase (GAA). Previously, we showed that adult muscle stem cells termed satellite cells are present at normal levels in muscle from patients with Pompe disease, but that these are insufficiently activated to repair the severe muscle pathology. Here we characterized the muscle regenerative response during disease progression in a mouse model of Pompe disease and investigated the intrinsic capacity of *Gaa*^*−*/−^ satellite cells to regenerate muscle damage. *Gaa*^−/−^ mice showed progressive muscle pathology from 15 weeks of age as reflected by increased lysosomal size, decreased fiber diameter and reduced muscle wet weight. Only during the first 15 weeks of life but not thereafter, we detected a gradual increase in centrally nucleated fibers and proliferating satellite cells in *Gaa*^−/−^ muscle, indicating a mild regenerative response. The levels of Pax7-positive satellite cells were increased in *Gaa*^−/−^ mice at all ages, most likely as result of enhanced satellite cell activation in young *Gaa*^−/−^ animals. Surprisingly, both young and old *Gaa*^−/−^ mice regenerated experimentally-induced muscle injury efficiently as judged by rapid satellite cell activation and complete restoration of muscle histology. In response to serial injury, *Gaa*^−/−^ mice also regenerated muscle efficiently and maintained the satellite cell pool. These findings suggest that, similar to human patients, *Gaa*^−/−^ mice have insufficient satellite cell activation and muscle regeneration during disease progression. The initial endogenous satellite cell response in *Gaa*^*−/−*^ mice may contribute to the delayed onset of muscle wasting compared to human patients. The rapid and efficient regeneration after experimental muscle injury suggest that *Gaa*^*−/−*^ satellite cells are functional stem cells, opening avenues for developing muscle regenerative therapies for Pompe disease.

**Electronic supplementary material:**

The online version of this article (10.1186/s40478-018-0620-3) contains supplementary material, which is available to authorized users.

## Introduction

Pompe disease is a metabolic myopathy that is caused by deficiency of acid alpha glucosidase (GAA), a lysosomal enzyme responsible for the degradation of glycogen [38]. Pompe patients develop progressive skeletal muscle weakness due to lysosomal expansion, followed by lysosomal disruption and myofiber death. Affected muscles include those involved in mobility and respiration, and as a result Pompe patients become wheelchair and ventilator dependent [59]. The most severe classic infantile form of Pompe disease is caused by complete absence of GAA enzyme activity and results in death within the first year of life, if left untreated [52]. In milder forms of Pompe disease, residual GAA activity exists, and patients develop symptoms later in life [17, 54]. A treatment for Pompe disease is available in the form of enzyme replacement therapy (ERT). ERT improves muscle function and prolongs survival [2, 4, 21, 24, 33, 36, 37, 51, 57], but the heterogeneous response among patients has urged the development of alternative treatment options [5].

Skeletal muscle has the capacity to regenerate upon damage. Genetic ablation of Pax7-expressing cells in mice has shown that this process is dependent on adult muscle stem cells termed satellite cells [25, 40]. In healthy muscle, satellite cells reside in a quiescent state located in between the sarcolemma and the basal lamina [30]. Upon muscle damage, satellite cells become activated and enter the cell cycle. Proliferating satellite cells have two fates: to repair muscle fibers, or to replenish the satellite cell pool [7]. Given the regenerative properties of skeletal muscle a major unresolved question in the field remains why satellite cells are apparently unable to efficiently repair disease-induced muscle damage. Several explanations have been proposed, including exhaustion of the satellite cell pool [39] or intrinsic failure of satellite cells to regenerate muscle [3, 10]. For example, in Duchenne Muscular Dystrophy, both satellite cell depletion/exhaustion and intrinsic failure of satellite cells to regenerate have been proposed [10, 39]. Previously, we have analyzed muscle biopsies from patients with Pompe disease [41]. Our study demonstrated a lack of muscle regeneration to the severe damage observed in biopsies from Pompe patients, even in those from severely affected classic infantile patients. We found that satellite cells were present at similar levels as in healthy controls, arguing against satellite cell depletion in Pompe disease [41]. However, satellite cells were mostly inactive, in agreement with the lack of detectable muscle regeneration.

The maintenance of the satellite cell pool in patients with Pompe disease suggested the possibility that endogenous satellite cells represent a therapeutic target for Pompe disease. A prerequisite for this idea is that satellite cells are intrinsically capable of regenerating muscle. So far, this remained unclear given the low level of muscle regeneration in Pompe patients. To address this, in the present study we used two knockout mouse models for Pompe disease on different genetic backgrounds [6, 35]. We characterized the satellite cell response in *Gaa*^−/−^ mice, and related this to myofiber pathology. We then applied a single and serial external injury and characterized muscle regeneration and the satellite cell response. Our results indicate that *Gaa*^−/−^ mice have activated satellite cells and low levels of muscle regeneration only during the first 15 weeks of life. Single and serial experimentally induced muscle injuries provoked efficient satellite cell activation and muscle regeneration in *Gaa*^−/−^ mice. These results indicate that satellite cells in *Gaa*^−/−^ mice have the intrinsic capacity to efficiently regenerate muscle and self-renew. These findings suggest that satellite cell activation may be explored as a therapeutic strategy to promote muscle regeneration in Pompe disease.

## Material and methods

### Mice and animal procedures

Age-matched wildtype and *Gaa*^−/−^ animals on an FVB/N [6] inbred, or mixed C57/Bl6 and 129/Sv [35] background were used between 2 and 70 weeks of age. Wild type FVB/N breeder animals were obtained from Envigo, and were used to start a colony that is maintained at the Erasmus MC animal facility. *Gaa*^−/−^(FVB/N) animals had been generated previously by targeted disruption of exon 13 of the *Gaa* gene [6]. We performed homozygous breedings to generate both the wildtype and *Gaa*^*−/−*^ animals in the FVB/N background during the duration of this project. Wildtype control and *Gaa*^*−/−*^ animals in the mixed C57/Bl6 and 129/Sv background were obtained as littermates from heterozygous breedings and maintained at the Cardarelli Hospital’ s Animal Facility (Naples, Italy). Gaa^−/−^(Bl6) animals obtained by insertion of a neo cassette into exon 6 of the *Gaa* gene [35] were purchased from Charles River Laboratories (Wilmington, MA). All mice in experiment were housed under a light–dark cycle (12 h) and under defined pathogen-free conditions, with access to food and water ad libitum.

Muscle injury was induced by intramuscular injection of 1.2% (*w*/*v* in PBS) BaCl_2_ or cardiotoxin (CTX; 10 μmol in PBS). Animals were allowed to recover for the time indicated in the figures. Serial injury experiments were performed by injecting BaCl_2,_ as described above, three times at monthly intervals into the Tibialis Anterior (TA) muscle. Three weeks after the last BaCl_2_ injection the animals were sacrificed for tissue collect.

At the end of experiments animals were sacrificed by cervical dislocation during daytime without a fixed timepoint. Tissue wet weight was determined by weighing freshly dissected tissue that was blotted dry. All animal experiments were approved by the local and national animal experiment authorities in compliance with the European Community Council Directive guidelines (EU Directive 86/609), regarding the protection of animals used for experimental purposes, and according to Institutional Animal Care and Use Committee (IACUC) guidelines for the care and use of animals in research. The study was approved by the local and national authorities in the Netherlands and Italy, respectively. All procedures with the animals were performed with the aim of ensuring that discomfort, distress, pain, and injury would be minimal.

### Determination of glycogen levels

To measure tissue glycogen concentrations 20 30 μm cryosections were collected for each sample. The sections were homogenized using 5 mm stainless steel beads (Qiagen NV) in the Qiagen Retsch MM300 TissueLyser (Qiagen NV) at 30 Hz for 5 min. Glycogen was quantified in tissue supernatant by measuring the amount of glucose released from glycogen after conversion by amyloglycosidase and amylase (Roche Diagnostics) for 1 h as previously described [58]. Spectral absorbance of the products was measured on a Varioskan spectrometer (Thermo Scientific) at 414 nm. Results from the glycogen measurements were normalized for protein content using the Pierce BCA protein assay kit (Thermo Scientific).

### Histology and immunofluorescent analyses

Hematoxylin and Eosin (HE) staining and Masson’s trichrome staining were performed using routine histology protocols as described previously [41]. For immunostaining, Tissue-Tek OCT-embedded tissue was snap-frozen in liquid nitrogen-cooled isopentane. 10 μm cryosections were cut and fixated in ice-cold aceton. A heated antigen retrieval procedure with 10 mM citrate buffer was used for the detection of Pax7. Sections were stained essentially as described previously [41], but using the M.O.M. kit from Vector laboratories for blocking endogenous mouse immunogens. Primary antibodies used were eMyHC (F1.652; DSHB; 1:300), Ki67 (Ab15580; Abcam; 1:50), laminin (L9393; Sigma; 1:500 or LS-C (6142; LS BIO; 1:500)), Lamp1 (Ab24170; Abcam; 1:150). Hoechst (H33258, Sigma) was used at 1 μg/ml. To detect centrally nucleated myofibers aceton-fixed 10 μm cryosections were stained for laminin using a primary antibody and Hoechst for nuclei, as described above, and imaged by fluorescent microscopy.

### Image acquisition and analysis

Histological sections were scanned with 4x and 20x objectives on a Hamamatsu NanoZoomer 2.0 (Hamamatsu Photonics). Images were analyzed using NDP view software (NDP View 1.2.31 Eng, Hamamatsu Photonics). Sections used for immunofluorescence were scanned on Zeiss LSM700 (Carl Zeiss B.V.) using tile-scan modality with a 20x objective. Image analysis and processing was performed using Fiji (fiji.sc/Fiji) and Adobe Photoshop. Quantification of myofiber diameter was performed using cross sections by measuring the longest diagonal (in μm) in at least 100 fibers per sample, randomly selected throughout the whole section.

### Flow cytometry

Preparation of limb muscle for flow cytometric analysis was adapted from Liu et al. [28]. In short, dissected tissue was minced thoroughly to small pieces in F10 medium (Lonza) containing collagenase II (750 U/ml; Fisher Scientific) using scalpels. Minced tissue was dissociated for 70 min in F10 medium containing 750 U/ml collagenase II), then for 30 min in F10 medium containing collagenase II (100 U/ml) and dispase (1.1 U/ml; Fisher Scientific). Cell suspensions were filtered over a 40 μM cell strainer (Falcon) and a small sample was retrieved to determine total mononuclear cell count using a hematocytometer. The cell suspensions were stained with CD31-APC (1:100), CD45-APC (1:100), Sca1-FITC (1:100) and Vcam-biotin (1:50) primary antibodies. Vcam was visualized using streptavidin-PECY7 (1:100). All antibodies for flowcytometry were derived from BD Biosciences. Cell viability was determined by staining with 1 μg/ml Hoechst 33258 (Sigma). Samples were analyzed on a BD-ARIAIII (BD biosciences).

### Statistical analysis

For all experiments normal distribution of data was determined based on calculated residuals. Normally distributed data from two groups was tested using a 2-tailed t-test. For experiments with three groups or more a one-way ANOVA of independent samples with Tukey or Games-Howell Post Hoc multiple correction (depending on homogeneity of variance) was used. Non-normal distributed data was statistically tested using a Mann-Whitney non-parametric test (two groups) or a Kruskal-Wallis test of independent samples with Bonferoni multiple comparison for three or more groups. For all tests a *p*-value less than 0.05 was considered significant. Data was analyzed using IBM SPSS statistics version 25.

## Results

### Characterization of muscle pathology during disease progression in *Gaa*^−/−^ mice

Previous work has shown that *Gaa*^−/−^ non-inbred mice (on FVB or C57/Bl6 backgrounds mixed with the 129 background) display hallmarks of Pompe disease, including progressive skeletal muscle wasting and glycogen accumulation. We now performed a more in-depth quantitative analysis of the timing of myofiber pathology and muscle wasting in FVB inbred mice (indicated as Gaa^−/−^(FVB)). Key results throughout this report were confirmed on *Gaa*^−/−^ mice in the C57/Bl6 non-inbred background (indicated as *Gaa*^−/−^(Bl6)).

**Fig. 1:**
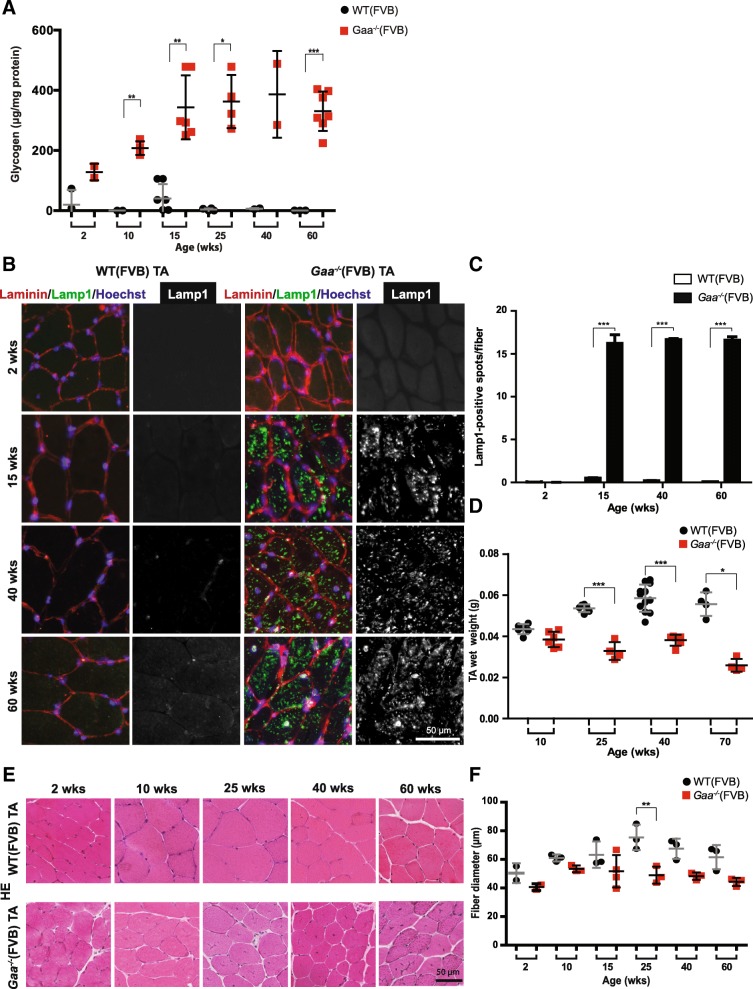
Characterization of lysosomal and muscle wasting pathology during disease progression in *Gaa*^−/−^ mice. **a**. Glycogen accumulation. Glycogen levels were measured biochemically in TA muscles at the indicated ages. **b**. Lysosomal pathology. Immunofluorescent analysis of TA sections using a Lamp1 antibody (in green). Representative images are shown. The basal lamina was stained using a Laminin antibody (in red). Nuclei were stained with Hoechst (in blue). Black and white images of Lamp1 staining are included for better visualization. **c**. Quantification of the number of Lamp1-positive spots per fiber from B. Data are from two TA muscles derived from two different animals per genotype per timepoint, and are expressed as mean ± SD. ***p < 0.001. **d**. Wet weight of TA muscles. Each dot represents TA wet weight from one muscle of one animal. Means ± SD are indicated as lines (*n* = 4–12 animals per genotype per timepoint). *p < 0.05 and ***p < 0.001. **e**. HE staining of TA sections. Representative images are shown. **f**. Quantification of fiber size from E. Data from individual mice are plotted (*n* = 2–4 animals per genotype per timepoint). Means ± SD are indicated. *p < 0.05 and **p < 0.01

Glycogen accumulation was detected in Tibialis Anterior (TA) muscle from *Gaa*^−/−^ mice at the age of 2 weeks, and increased further during aging (Fig. 1a). Maximal glycogen levels were reached in animals of about 25 weeks and remained stable thereafter. To examine abnormalities in lysosomal size, immunofluorescent staining of Lamp1 was performed (Fig. 1b). No Lamp1 staining was detected in TA muscle sections from wild type mice due to the small size of lysosomes. In *Gaa*^−/−^ muscle, TA sections showed a punctated Lamp1 pattern from 15 weeks of age and onwards indicating increased lysosomal size. The number of Lamp1-positive spots per fiber did not increase further in older *Gaa*^−/−^ mice (Fig. 1b-c). TA wet weight was reduced in *Gaa*^−/−^ mice compared to wild type mice, starting at 25 weeks of age, and decreased further with age with 54% at 70 weeks of age (Fig. 1d). Histological analysis of TA sections showed a decrease in fiber diameter in the *Gaa*^−/−^ mice compared to wild type mice that started between 15 and 25 weeks of age (Fig. 1e-f). Fiber diameter distribution was determined by quantifying the number of fibers in different fiber size categories. This showed enrichment of smaller-sized fibers in muscles of 15-week *Gaa*^−/−^ muscles relative to those from age-matched wild type animals (Additional file 1: Figure S1A). *Gaa*^−/−^ Quadriceps Femoris (QF) muscles at 3 months of age in the C57/Bl6 non-inbred background also displayed enrichment for smaller fibers (Additional file 1: Figure S1B). These results indicate that *Gaa*^−/−^(FVB) mice show progressive myofiber pathology reminiscent of Pompe disease starting at 15 weeks of age.

### Modest and transient muscle regeneration during disease progression in *Gaa*^−/−^ mice

**Fig. 2:**
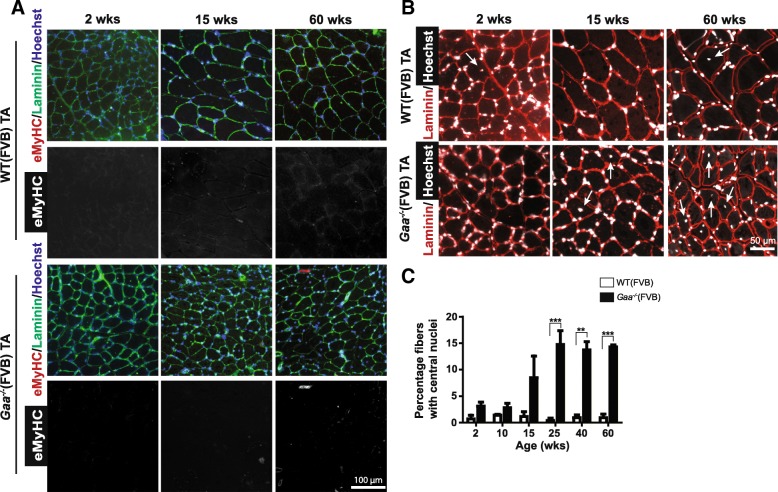
*Gaa*^−/−^ mice display modest and transient muscle regeneration during disease progression. **a**. eMyHC expression. Immunofluorescent staining of TA sections using a MyHC antibody (in red). Representative images are shown. The basal lamina was stained using a Laminin antibody (in green). Nuclei were stained with Hoechst (in blue). Black and white images of eMyHC staining are included for better visualization. **b**. Central nucleated fibers. Representative images of TA sections stained with Laminin (in red) and Hoechst (in white). **c**. Quantification of central nucleated fibers from B. Data represent means ± SD (*n* = 2–3 muscles from at least 2 different animals per genotype per timepoint). *p < 0.05. **p < 0.01 and ***p < 0.001

To assess whether *Gaa*^−/−^ (FVB) mice regenerate TA muscle in response to Pompe disease-induced muscle pathology, we first performed immunofluorescent staining of embryonic Myosin Heavy Chain (eMyHC), a marker for actively regenerating myofibers [42] (Fig. 2a). Few small-sized eMyHC-positive fibers were detected in muscles from *Gaa*^−/−^ animals between 2 and 60 weeks of age (< 0.7% of myofibers). Representative examples are shown in Fig. 2a. A similar result was obtained for *Gaa*^−/−^ animals in the C57/Bl6 non-inbred background (Additional file 2: Figure S2A). Muscle from wild type mice did not show eMyHC-positive fibers in either background.

In murine muscle, regenerated myofibers can be identified by the presence of centrally located nuclei [27]. We used Hoechst to stain nuclei in TA sections (Fig. 2b), and this showed an increased percentage of fibers with central nuclei in *Gaa*^−/−^ TA muscle from 15 weeks of age (Fig. 2c). At 25 weeks of age, the percentage of centrally nucleated fibers reached a plateau of 15% that remained stable until 60 weeks of age. In comparison, published results in the mdx mouse, a model for Duchenne Muscular Dystrophy, showed that already at 12 weeks of age > 70% of lower limb muscle myofibers were centrally nucleated [1], indicating that the disease-mediated muscle-regenerative response is relatively mild in *Gaa*^−/−^ muscle. Modest central nucleation was also detected in GAS muscle at 3 month-old *Gaa*^−/−^(Bl6) mice (Additional file 2: Figure S2B). We conclude that *Gaa*^−/−^ mice have a relatively mild and limited muscle regenerative response during disease progression.

### Satellite cells are increased in number but are only transiently activated during disease progression in *Gaa*^−/−^ limb muscle

**Fig. 3:**
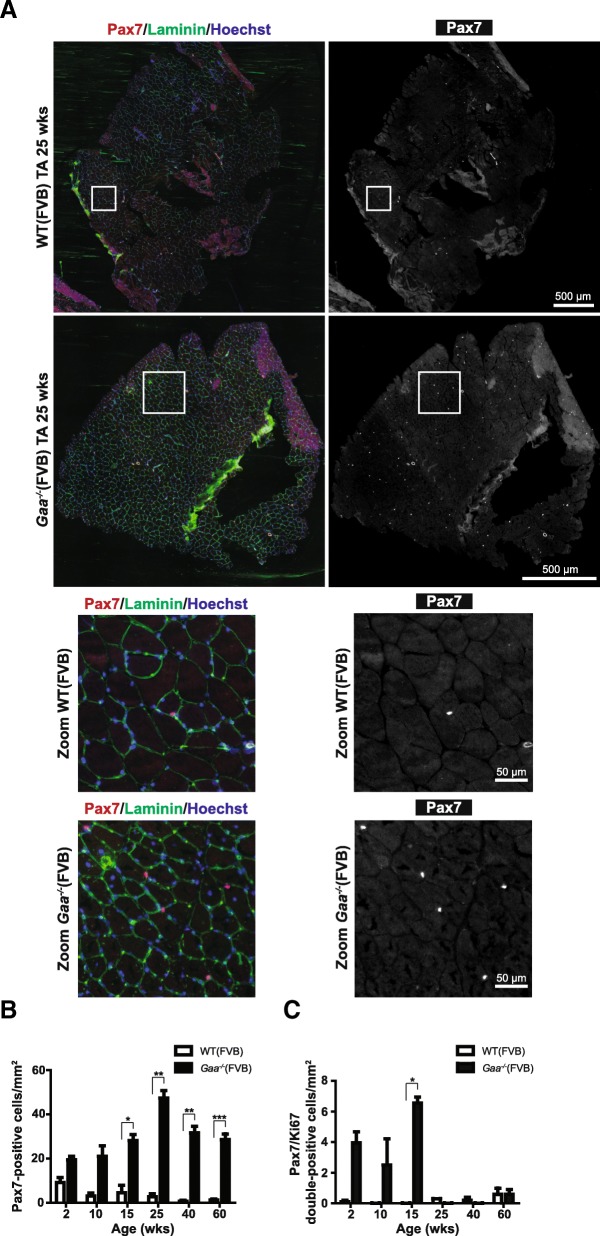
Satellite cells are increased in number but are only transiently activated during disease progression in *Gaa*^−/−^ limb muscle. **a**. *pax*7 expression. Immunofluorescent (IF) staining of TA sections using a Pax7 antibody (in red). Representative images are shown. The basal lamina was stained using a Laminin antibody (in green). Nuclei were stained with Hoechst (in blue). Black and white images of Pax7 staining are also shown for better visualization. Zooms of selected areas (white squares) are shown below the entire sections. **b**. Quantification of the number of Pax7-positive cells/mm^2^ from A. Data are means ± SD from 2 muscles derived from 2 different animals per genotype per timepoint. *p < 0.05. **p < 0.01 and ***p < 0.001. **c**. Quantification of the number of Pax7/Ki67 double-positive cells by immunofluorescent staining of TA sections using Pax7 and Ki67 antibodies. Representative stainings are shown in Additional file 5: Figure S5. Data represent means ± SD from 2 TA muscles derived from 2 different animals. *p < 0.05

Genetic ablation of Pax7-expressing cells demonstrated that satellite cells are indispensable for muscle regeneration [25, 40]. Satellite cells are marked by expression of Pax7, which is a master transcription factor that regulates survival and expression of myogenic transcription factors involved in muscle differentiation and regeneration [23, 43]. To assess the consequence of Gaa-deficiency and the related muscle pathology on satellite cell dynamics, we performed immunofluorescent staining of Pax7 in TA sections (Fig. 3a). The number of Pax7-positive cells was stably increased in *Gaa*^−/−^ TA muscle relative to wild type muscle at all ages tested (2–60 weeks), and varied between 20 and 50 Pax7-positive cells/mm^2^ (Fig. 3b). The increase in Pax7-positive cells in *Gaa*^−/−^ muscle was equally pronounced when expressed as satellite cell per myofiber, with a ~ 5 fold increase at 15 weeks and ~ 7.1 fold increase at 25 week animals (Additional file 3: Figure S3), indicating that the difference in satellite cell density was independent of changes in fiber diameter.

The number of Pax7-positive cells in wild type TA muscle decreased from 8 to 2 Pax7-positive cells/mm^2^ during the same period (Fig. 3b). To confirm increased satellite cell levels in *Gaa*^−/−^ mice, we analysed the number of satellite cells by flow cytometry using a satellite cell surface profile based on expression of Vcam [28] (Additional file 4: Figure S4A). Using this profile we could detect a > 93% pure population of Pax7-positive cells (Additional file 4: Figure S4B). The number of Vcam-positive cells was stably increased in *Gaa*^−/−^ TA muscle between 15 and 70 weeks of age relative to wild type TA muscle (Additional file 4: Figure S4C). Satellite cell numbers were also increased in *Gaa*^−/−^muscle in the C57/Bl6 non-inbred background as shown by immunofluorescent analysis of Pax7-positive cells in 3 months old gastrocnemius (GAS) muscles from WT(Bl6) and *Gaa*^−/−^(Bl6) mice (Additional file 5: Figure S5A-B). We also detected increased expression of Pax7 protein by immunoblotting of *Gaa*^−/−^(Bl6) GAS muscle (Additional file 5: Figure S5C).

Upon activation, satellite cells proliferate in order to regenerate muscle and replenish the satellite cell pool. To assess whether satellite cells in *Gaa*^−/−^ mice were activated in response to muscle pathology, we analysed co-expression of Pax7 and the proliferation marker Ki67 by immunofluorescent analysis (Additional file 6: Figure S6). In TA muscles from young *Gaa*^−/−^ mice up to 15 weeks of age ± 5% of satellite cells were Ki67-positive, while in older *Gaa*^−/−^ mice the Pax7-positive cells were Ki67-negative (Fig. 3c). In wild type mice, Pax7-positive cells were Ki67-negative at all ages tested. These results indicate that while satellite cell numbers were increased in *Gaa*^−/−^ mice of all ages, satellite cell activation was only observed during the first 15 weeks. In older mice activated satellite cells were absent.

### *Gaa*^−/−^ mice efficiently regenerate muscle after experimental injury

**Fig. 4:**
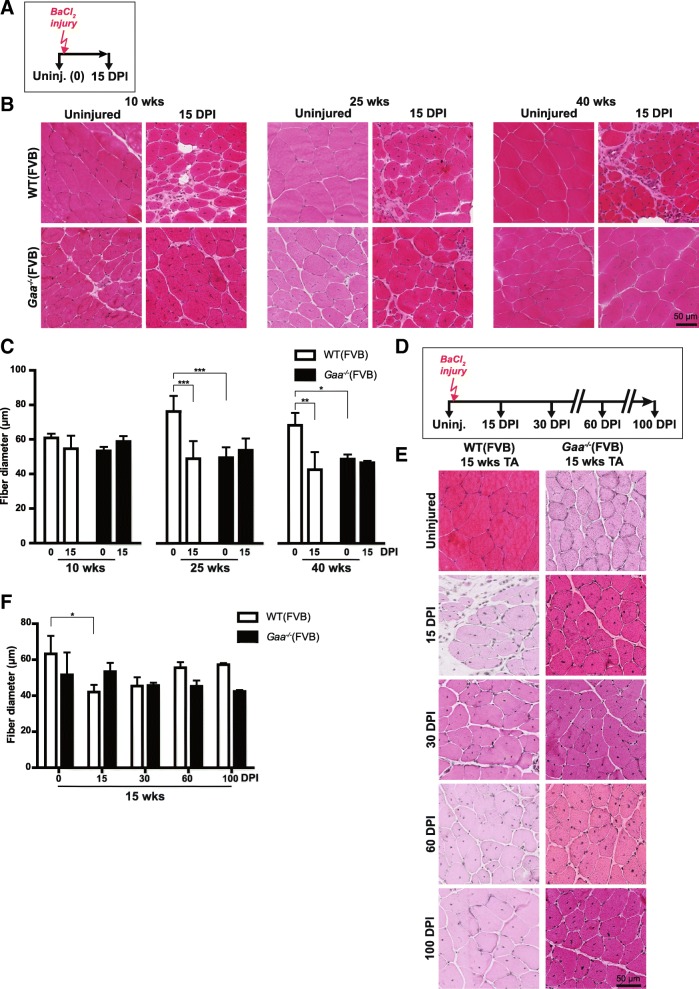
*Gaa*^−/−^mice regenerate muscle efficiently after experimental injury. **a**. Schematic representation of the injury experiment. Black arrows indicate the time at which TA muscles were collected for analysis, the red arrow indicates the time of injury. **b**. HE staining of TA sections before (Uninjured, 0 days post injury (DPI)) and at 15 days DPI with BaCl_2_ at three ages. Representative images are shown. **c**. Quantification of fiber diameter from (**b**). **d**. Schematic representation of injury experiment with a longer follow up after injury. Black arrows indicate the time at which TA muscles were collected for analysis, red arrow indicate the time of injury. **e**. HE staining of TA sections of the injury experiment with long follow-up. Representative images are shown. **f**. Quantification of fiber diameter from E. Data in C and F are means ± SD from at least 3 muscles derived from 2 or more different animals. *p < 0.05; **p < 0.01 and ***p < 0.001

Similar to human Pompe patients, *Gaa*^−/−^ mice insufficiently regenerated disease-induced muscle damage. To determine whether this is an intrinsic property of *Gaa*^−/−^ satellite cells or caused by compromised satellite cell activation, we induced muscle injury using BaCl_2_ injection (Fig. 4a). BaCl_2_ induces myofiber degeneration but leaves satellite cells sufficiently unharmed to allow regeneration [18]. BaCl_2_ was injected into TA muscles in 10, 25, or 40 weeks-old mice, and muscle regeneration was examined at 15 days post injury (DPI) (Fig. 4a). *Gaa*^−/−^ mice from all three age groups efficiently regenerated TA muscle upon injury, as judged by histological analysis of HE-stained tissue sections (Fig. 4b). At 15 DPI, regeneration was completed in *Gaa*^−/−^ mice of all three age groups while the wild type muscle was still in the process of regenerating damage. Quantification of the fiber diameter before and after the injury confirmed these observations (Fig. 4c). To extend these results, we followed regeneration for up to 100 DPI using 15 week-old mice (Fig. 4d). As judged by morphology, muscle regeneration was complete in *Gaa*^−/−^ mice at 15 DPI in *Gaa*^−/−^ mice, and between 30 and 60 DPI in wild type mice (Fig. 4e). Quantification of fiber diameter confirmed these observations (Fig. 4f). To examine the development of chronic tissue fibrosis that may result from incomplete regeneration as is observed in dystrophic muscle [9], we performed trichrome staining on regenerated muscle. This showed some trichrome-positive areas at time points before regeneration was completed in animals of both genotypes reflecting the transient expansion of fibro/adipogenic progenitors that is part of normal muscle regeneration [20] (Additional file 7: Figure S7). At 15 DPI little or no trichrome staining was detected in *Gaa*^−/−^ or wild type mice at 10, 15, or 40 weeks of age (Additional file 8: Figure S8), suggesting successful tissue remodelling and absence of fibrotic muscle tissue replacement. In mice in the C57/Bl6 non-inbred background, injury was induced using cardiotoxin (CTX) injection into Quadriceps Femoris (QF) muscle at 3 months and 11 months of age, or into the GAS muscle at 3 months of age. All these tissues showed efficient regeneration in *Gaa*^−/−^ mice at similar efficiencies compared to wild type mice, as judged by morphology (Additional file 9: Figure S9). We conclude that *Gaa*^−/−^ mice have efficient intrinsic capacity to regenerate muscle after experimentally-induced injury.

### Satellite cell response in *Gaa*^−/−^ mice after experimental injury

**Fig. 5:**
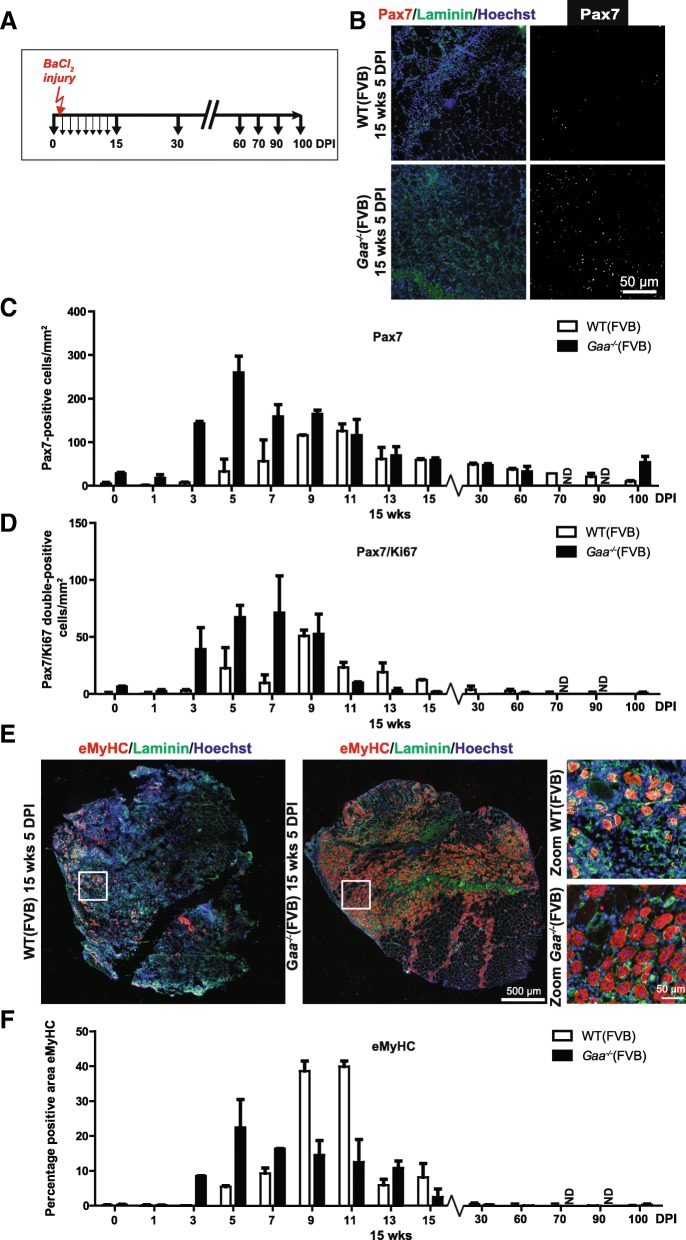
Rapid satellite cell response in *Gaa*^−/−^ mice after experimental injury. **a**. Experimental schedule. Black arrows indicate the time at which TA muscles were collected for analysis, the red arrow indicates the time of injury. **b**. *pax*7 expression. Immunofluorescent (IF) staining of TA sections using a Pax7 antibody (in red). Representative images are shown. The basal lamina was stained using a Laminin antibody (in green). Nuclei were stained with Hoechst (in blue). Black and white images of Pax7 staining are included for better visualization. **c**. Quantification of the number of Pax7-positive cells/mm^2^ from B. Data are means ± SD from at least 2 muscles from 2 different animals. **d**. As C, but now for the number of Pax7/Ki67 double-positive cells/mm^2^. **e**. eMyHC expression at 5 DPI. Immunofluorescent staining using a MyHC antibody (in red). Representative images are shown. The basal lamina was stained using a Laminin antibody (in green). Nuclei were stained with Hoechst (in blue). Zooms of selected areas (white squares) are shown on the right. **f**. Quantification of the eMyHC-positive area in TA sections. Data are means ± SD from at least 2 muscles derived from 2 different animals

To determine whether the efficient muscle regeneration upon induced injury in *Gaa*^−/−^ mice is accompanied by satellite cell activation, we quantified the number of Pax7-positive and Pax7/Ki67 double-positive cells at several time points following BaCl_2_ injection in the TA muscle (Fig. 5a-b). In both *Gaa*^−/−^ and wild type mice, the number of Pax7-positive cells was transiently and strongly induced upon injury. In *Gaa*^−/−^ mice, satellite cell numbers were already increased at 3 DPI, peaked at 5 DPI, and then slowly returned to pre-injury numbers during 7–60 DPI (Fig. 5c). Wild type mice showed slightly slower kinetics, with increased satellite cell numbers that started increasing at 5 DPI and peaked at 9–11 DPI, and slowly returned to pre-injury levels during 11–100 DPI. The kinetics of the number of Pax7/Ki67 double-positive cells paralleled those of Pax7-single positive cells in both *Gaa*^−/−^ and wild type mice (Fig. 5d).

In the same experiment, active muscle regeneration was examined using immunofluorescent staining of eMyHC (Fig. 5e). The kinetics of eMyHC expression in *Gaa*^−/−^ mice paralleled the changes in number of Pax7-expressing cells after injury (Fig. 5f; compare with Fig. 5c). Together, these data show that the efficient regenerative response of *Gaa*^−/−^ muscle after BaCl_2_-induced injury is mediated by a rapid satellite cell response and further confirms that *Gaa*^−/−^ satellite cells are not functionally compromised.

### *Gaa*^−/−^ satellite cells regenerate muscle and self-renew after serial injury

**Fig. 6:**
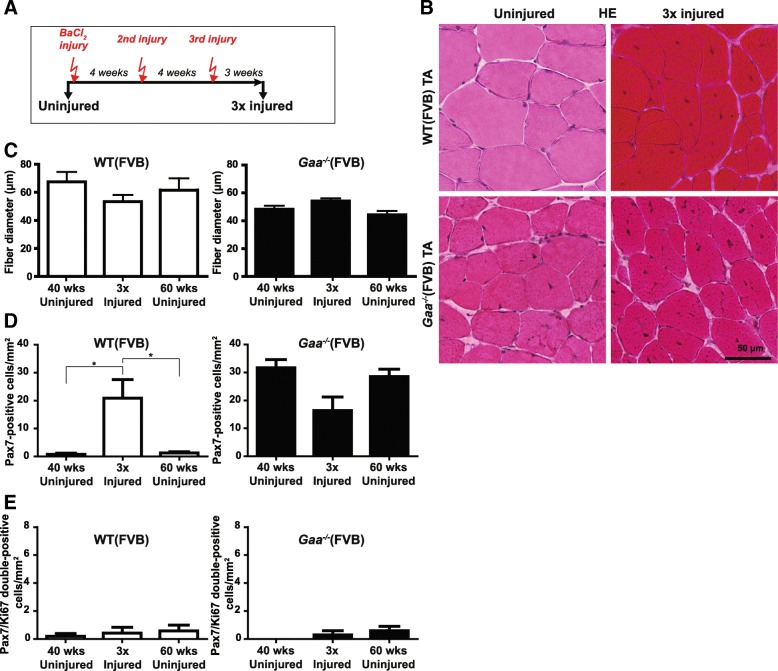
*Gaa*^−/−^ satellite cells regenerate muscle and self-renew after serial injury. **a**. Experimental schedule. Black arrows indicate the time at which TA muscles were collected for analysis, red arrows indicate the time of injury. **b**. HE staining of TA sections before and 3 weeks after the third injury. Representative images are shown. **c**. Quantification of fiber diameter in WT (*left*) and *Gaa*^−/−^ (*right*) TA muscle after serial injury from (**b**). **d**. Quantification of the number of Pax7-positive cells by immunofluorescent staining of TA sections using a Pax7 antibody in WT (*left*) and *Gaa*^−/−^ (*right*) TA muscle after serial injury. **e**. Quantification of the number of Pax7/Ki67 double-positive cells by immunofluorescent staining of TA sections using Pax7 and Ki67 antibodies in WT (*left*) and *Gaa*^−/−^ (*right*) TA muscle after serial injury. Data in **c**-**e** are means ± SD from at least 2 muscles derived from 2 different animals. For comparison with levels of indicated parameter in uninjured age-matched TA muscles, values at 40 weeks of age (to compare with the start of the experiment) and at 60 weeks of age (to compare with the end of the experiment) are included in (**c**-**e**)

*Gaa*^−/−^ mice showed efficient satellite cell-mediated muscle regeneration upon a single induced injury. To determine if *Gaa*^−/−^ satellite cells can self-renew after injury, which is essential for long-term muscle regeneration, we performed a serial injury experiment using BaCl_2_ (Fig. 6a). Three consecutive injuries were applied at 4 week intervals to the same TA muscles and animals were allowed to regenerate in between injuries. At the end of the experiment, 3 weeks after the last injury (mice were 51 weeks of age at this timepoint), mice were sacrificed and TA muscles were analysed. Histological analysis of HE-stained tissue sections showed complete regeneration from the serial injuries in both *Gaa*^−/−^ and wild type muscle (Fig. 6b). Quantification of fiber diameter showed full restoration of fiber diameter in *Gaa*^−/−^ mice 3 weeks after the third injury (Fig. 6c). In wild type mice, fiber diameter was not yet fully restored at this time point, consistent with the slower kinetics after a single injury (Fig. 6c, see also Fig. 4f). Therefore the parameters of this experiment did not allow to fully assess the capacity of wild type mice to regenerate after serial injury. The number of Pax7-positive cells in three-times regenerated *Gaa*^−/−^ muscle was, although slightly lower, not significantly different from satellite cell levels in pre-injury muscles or in muscles at 60 weeks of age (Fig. 6d). In wild type mice, the number of Pax7-positive cells was still enhanced at 3 weeks after the third injury, in line with the slower regeneration kinetics after a single injury (see Fig. 4f). The levels of Pax7/Ki67 double-positive cells at 3 weeks after the third injury were very low in both *Gaa*^−/−^ and wild type TA muscle, consistent with their levels at 3 weeks after a single injury (compare with Fig. 5d). This showed that also after repeated injury, satellite cells in *Gaa*^−/−^ TA muscle returned within a normal timeframe to their quiescent state. We conclude that *Gaa*^−/−^ mice have a robust capacity to regenerate muscle via satellite cells even after repeated injury and that *Gaa*^−/−^ satellite cells retain the capacity to self-renew upon injury.

## Discussion

In this study, we have used mouse models for Pompe disease to assess the muscle regenerative capacity of satellite cells. We first determined the timing of muscle pathology, and found the following sequence of events: glycogen accumulation (starting at 2 weeks), enlarged lysosomes (starting at 15 weeks of age), reduced fiber diameter (starting at 15–25 weeks of age), and reduced wet weight (starting at 25 weeks of age). Gaa-deficient mice display a mild muscle regenerative response shortly after birth up to 25 weeks of age, indicated by a gradual increase in central nucleation, detection of some eMyHC-positive myofibers and low-level satellite cell activation. This correlated with the detection of proliferating satellite cells during this period, but not thereafter. Satellite cell proliferation during the first 15 weeks of age resulted in stably increased levels of satellite cells in animals up to at least 60 weeks of age. Induced muscle injury in *Gaa*^*−/−*^ mice using BaCl_2_ or CTX resulted in very efficient satellite cell response and muscle regeneration. In addition, *Gaa*^*−/−*^ muscle regenerated completely after three consecutive rounds of injury and regeneration, indicating that *Gaa*^−/−^ satellite cells are capable of self-renewal. These results indicate that, similar to human Pompe patients, *Gaa*^*−/−*^ mice lack an efficient muscle regenerative response during disease progression but maintain the satellite cell pool despite the developing muscle damage. Importantly, satellite cells in mice with Pompe disease have the intrinsic capacity to efficiently regenerate after damage, suggesting that the lack of a satellite cell response in Pompe disease is caused by deficient satellite cell activation.

The mouse models for Pompe disease offers the opportunity to study the early stages of disease onset and to link these to the muscle regenerative response. We have used an inbred FVB strain as well as *Gaa*^−/−^ mice on a mixed C57/Bl6 and 129/Sv background to investigate this. The key aspects of muscle regeneration activity in Gaa-deficient muscle were observed in both these mouse models, including the mild regenerative response during disease progression, reflected by the gradual increase in central nucleation and detection of few eMyHC-expressing myofibers, together with increased satellite cell levels and an efficient regenerative response after experimental injury. This strengthens the conclusion that the regenerative response during Pompe disease progression is inefficient and disturbed. To address this point in more detail, we used *Gaa*^*−/−*^ animals in the FVB background to extensively characterize the regeneration response as well as the ability to regenerate after (serial) experimental injury. This extended on earlier reports [6, 35] that the *Gaa*^*−/−*^ mouse models develops symptoms more slowly compared to classic infantile Pompe patients, even though in both human and mouse cases, Gaa activity was completely disrupted. Classic infantile Pompe patients show symptoms shortly after birth [55] and these include generalized muscle weakness evident by decreased muscle tone and strength. Muscle biopsies from classic infantile Pompe patients show severely damaged muscle fibers [41, 48, 56]. At the same time, satellite cells in classic infantile patients are not activated and muscle regeneration is undetectable [41]. In contrast, *Gaa*^*−/−*^ mice developed cellular pathology at adulthood, starting at 15–25 weeks of age, as indicated by increased lysosomal size and decreased fiber diameter and wet weight. Interestingly, satellite cells in *Gaa*^*−/−*^ mice were activated until the age of 15 weeks, as indicated by the detection of Pax7-positive satellite cells that co-express Ki67. Proliferating satellite cells were not detected in older animals. This may suggest that the endogenous satellite cells response in the first 15 weeks after birth contributed to the delayed onset of muscle wasting in *Gaa*^*−/−*^ mice compared to that in human classic infantile patients. It is interesting to speculate that a modest satellite cells response and muscle regeneration activity may prevent the development of muscle fiber pathology. Future work is required to test this notion.

It remains unclear why the satellite cell response in Pompe disease is so modest (mouse) or not detectable (human). In certain other neuromuscular disorders, satellite cells and muscle regeneration have a markedly different behaviour. For example, in Duchenne Muscular Dystrophy, studies using the *mdx* mouse model or the more severe *mdx/utrophin* double-knockout model have reported both exhaustion and depletion of satellite cells as result of continuous satellite cell activation during disease progression [29, 39]. In muscle biopsies from Duchenne patients, elevated muscle regeneration activity is observed [8, 22, 31, 39, 41]. Increased satellite cell activation is attributed to sarcolemmal fragility as result of loss of functional dystrophin. Loss of sarcolemmal integrity triggers the release of signals from the muscle environment [62], from the damaged myofiber itself [16] or from other cell types that are recruited and activated after damage [19, 20]. These events promote the transient infiltration of immune cells that are essential for proper regeneration and participate in satellite cell activation [49]. However, chronic inflammation in dystrophic muscle contributes to disease progression and dysregulated satellite cell activation, as has also been proposed for inflammatory myopathies [61]. In Pompe disease the primary defect is not at the sarcolemma [15] and an aberrant immune response is generally absent in Pompe disease [41], indicating that satellite cell function and activation are differently regulated in Gaa-deficient muscle. It is tempting to speculate that the lysosomal damage as result of glycogen accumulation and/or the subsequent block of autophagy [13, 32] interfere with satellite cell activation. It has been established that autophagy is crucial for securing the bioenergetics demands associated with satellite cell activation [46]. Deficiency of *SIRT1*, a nutrient sensor that regulates autophagic flux in satellite cell progeny, was found to delay satellite cell activation [46]. Inhibition of autophagic flux has been reported to occur in *Gaa*^−/−^ myofibers [12, 36], but whether this affects satellite cell activation remains to be determined.

Satellite cells in *Gaa*^−/−^ muscle do not respond to the progressive tissue damage, in Pompe patients as well as in mice of 15 weeks and older. Our finding that experimentally-induced muscle injury evokes an efficient muscle regenerative response suggests that once satellite cells are activated, downstream processes such as myogenic differentiation and myoblast fusion are unaffected by *Gaa* deficiency. This is in agreement with the normal myogenic differentiation of induced pluripotent stem cells established from Pompe patients’ fibroblasts, even those generated from a severely affected classic infantile patient [60]. We speculate that the maintenance of satellite cell function and number, as well as a functional regeneration machinery offers opportunities for developing a muscle regenerative therapy for Pompe disease through stimulation of endogenous satellite cells. Satellite cells can be safely and efficiently activated through exercise [45]. Previous exercise programs in our and other centers were found to be well tolerated by and beneficial for adult Pompe patients [11, 26, 34, 44, 47, 50]. It can be predicted that induced satellite cell activation would be less favorable in untreated classic infantile patients, since these patients display severe pathology directly after birth, and newly regenerated muscle fibers likely develop pathology rapidly. However, treatment of classic infantile patients with ERT can significantly improve muscle function and morphology [53] and delay the severity of symptoms. This would suggest that combining ERT with induced satellite cell activation might be beneficial for these patients. The development of pathology can take years in patients with a more slowly progressing disease course. Based on our findings in the mouse model of Pompe disease, restoration of the muscle condition via induced regeneration would be predicted to delay disease progression. We consider it worthwhile to extend research on satellite cell activation in Pompe disease and other neuromuscular disorders that harbor functional yet inactive satellite cells. Identification of additional muscle diseases with such a profile may result in the development of a more generic therapeutic strategy. If successful, such strategy would be highly valuable given the scarcity of treatment options for neuromuscular disorders.

## Conclusion

The current study shows that in *Gaa*^−/−^ mice satellite cell activation and muscle regeneration was insufficient to repair the disease-mediated damage, similar as was observed in human patients. However, *Gaa*-deficient satellite cells were intrinsically capable of regenerating muscle and harbored self-renewal potential. Our findings suggest that the muscle phenotype in Pompe disease may be ameliorated by regenerative therapies directed at satellite cell activation.

## Additional files

Additional file 1: **Figure S1.** Reduced fiber diameter in GAAKO animals. A. Fiber diameter frequency distribution plot of WT(FVB) and *Gaa*^−/−^(FVB) at 15 weeks of age. B. WT(Bl6) and GAAKO(Bl6) at 3 months of age showing reduced fiber diameter in *Gaa*^−/−^ (Bl6). These data suggest muscle atrophy is observed in GAA-deficient animals on both FVB/N and C57/Bl6 backgrounds. (PDF 122 kb)

Additional file 2: **Figure S2.** Modest muscle regeneration in *Gaa*^−/−^(Bl6) animals. A.eMyHC staining of QF sections from WT(Bl6) and *Gaa*^−/−^(Bl6) animals. The figure shows selected areas of eMyHC (*red*)/laminin(*green*)/Hoechst(*blue*) stained QF sections from 4, 12 and 36 week old WT(Bl6) and *Gaa*^−/−^ (Bl6) animals. eMyHC-positive were rare and very small in *Gaa*^−/−^ (Bl6) muscle, in line with findings in *GAA*^−/−^(FVB) (see Fig. 2A). B. Examples of HE-stained section from GAS muscle from 3 months old WT(Bl6) and *Gaa*^−/−^(Bl6) animals. (PDF 320 kb)

Additional file 3: **Figure S3.** Satellite cell numbers are increased in *Gaa*^−/−^ TA muscle. The number of Pax7-positive cells in 15 week (A) and 25 week (B) WT and *Gaa*^−/−^ from Fig. 3 expressed as Pax7-positive cells/myofiber. Data are means ± SD from 2 muscles derived from 2 different animals per genotype per timepoint. *p < 0.05 and **p < 0.01. (PDF 103 kb)

Additional file 4: **Figure S4.** Identification of Pax7-positive satellite cells by flow cytometry. A. Representative dot plots from CD31-APC/CD45-APC/Sca1-FITC/Vcam-PeCY7 stained muscle cell suspensions according to the procedure described previously by Liu et al. [28]. The gating strategy is depicted by the green arrow. The colors of the box/plot outlines correspond with the gated populations. Satellite cells are in the CD45neg/CD31neg/sca1-neg/Vcam-positive gate (green box). *B. pax*7 (*red*)/Hoechst(*blue*) staining of FACS-sorted satellite cells after 24 h culture using the procedure shown in (A). The lower panel shows the zoom of the insert in the upper panel. Counting Pax7 expressing cells indicated that sorting was performed at > 93% purity. C. Quantification of the percentage of Vcam-positive cells by flow cytometry. Data from individual mice are plotted as single dots. (PDF 1056 kb)

Additional file 5: **Figure S5.** Satellite cell numbers are increased in *Gaa*^−/−^(Bl6) muscle. A. Satellite cells were detected in 3 months old WT(Bl6) and *Gaa*^−/−^(Bl6) gastrocnemius (GAS) cryosections by immunofluorescent staining of Pax7 (*red*). Myofibers were visualized using a laminin antibody (*green*) and nuclei with Hoechst (*blue*). White arrows point to Pax7-positive satellite cells. B. Quantification of A. The figure depicts the mean percentage of Pax7-positive Satellite cells per field ± SD. C. Western blot analysis of Pax7 expression in GAS muscle from 13 week old WT(Bl6) and *Gaa*^−/−^(Bl6) animals. Western blot analysis was performed as previously described [14]. (PDF 952 kb)

Additional file 6: **Figure S6.** Detection of proliferating satellite cells in GAA-deficient limb muscle. Representative images from TA limb muscle sections co-stained for Pax7 (*red*) and Ki67 (*green*) to detect proliferating satellite cells (arrow). Nuclei are visualized with Hoechst (*blue*). The arrowhead points to a Pax7-positive/Ki67-negative quiescent satellite cell. (PDF 286 kb)

Additional file 7: **Figure S7.** Detailed histological evaluation of regenerating WT and *Gaa*^−/−^ muscle. The figure depicts HE- and trichrome stained histological sections from 15 week WT(FVB) and *Gaa*^−/−^ (FVB) TA muscles at multiple time-points during the first 15 days after BaCl_2_-induced muscle regeneration. *Gaa*^−/−^ (FVB) muscle regenerates efficiently and completely (*left* panels). The trichrome stain shows absence of residual fibrotic tissue after completing a regeneration cycle (*right* panels). As explained in the text WT(FVB) has a regeneration cycle of more than 30 days and was therefore still actively remodelling at 15 DPI. Arrows point to small de novo myofibers detected as early as 3 DPI in regenerating *Gaa*^−/−^(FVB) muscle. (PDF 569 kb)

Additional file 8: **Figure S8.** *Gaa*−/− muscle regenerates completely without tissue remodeling Depicted are images from trichrome staining of TA muscle of WT(FVB) and *Gaa*^−/−^(FVB) at 10, 25 and 40 weeks before and 15 days after BaCl_2_ injury. (PDF 348 kb)

Additional file 9: **Figure S9.** Efficient regeneration of GAAKO-muscle after cardiotoxin-induced injury in GAA-deficient animals on a C57/Bl6 background. The figure shows histological sections from TA HE-stained sections from 12 and 48 week old WT(Bl6) and *Gaa*^−/−^ (Bl6) animals at indicated time points after injury uisng cardiotoxin (CTX)-injection. The *upper* panels show HE-stained sections from QF muscle, while the *middle* panels show images from regenerating GAS. The *lower* panels depict regenerating QF from 11 months old WT(Bl6) and *Gaa*^−/−^ (Bl6) animals. These data demonstrate that the capacity to regenerate after experimental injury is also maintained in GAA-deficient muscle on a C57/Bl6 background. (PDF 386 kb)

## Abbreviations

BaCl_2_: Barium chloride
CNF: Central nucleated fibers
CTX: Cardiotoxin
DPI: Days post injury
eMyHC: Embryonic myosin heavy chain
ERT: Enzyme replacement therapy
GAA: Acid alpha glucosidase
GAS: Gastrocnemius
OCT: Optimal cutting temperature
PFA: Paraformaldehyde
QF: Quadriceps Femoris
TA: Tibialis Anterior
