# Supplementary figures and images for: Satellite cells maintain regenerative capacity but fail to repair disease-associated muscle damage in mice with Pompe disease
Source: Acta Neuropathol Commun. 2018 Nov 7;6:119. doi: 10.1186/s40478-018-0620-3 (PMC6220463; doi:10.1186/s40478-018-0620-3)

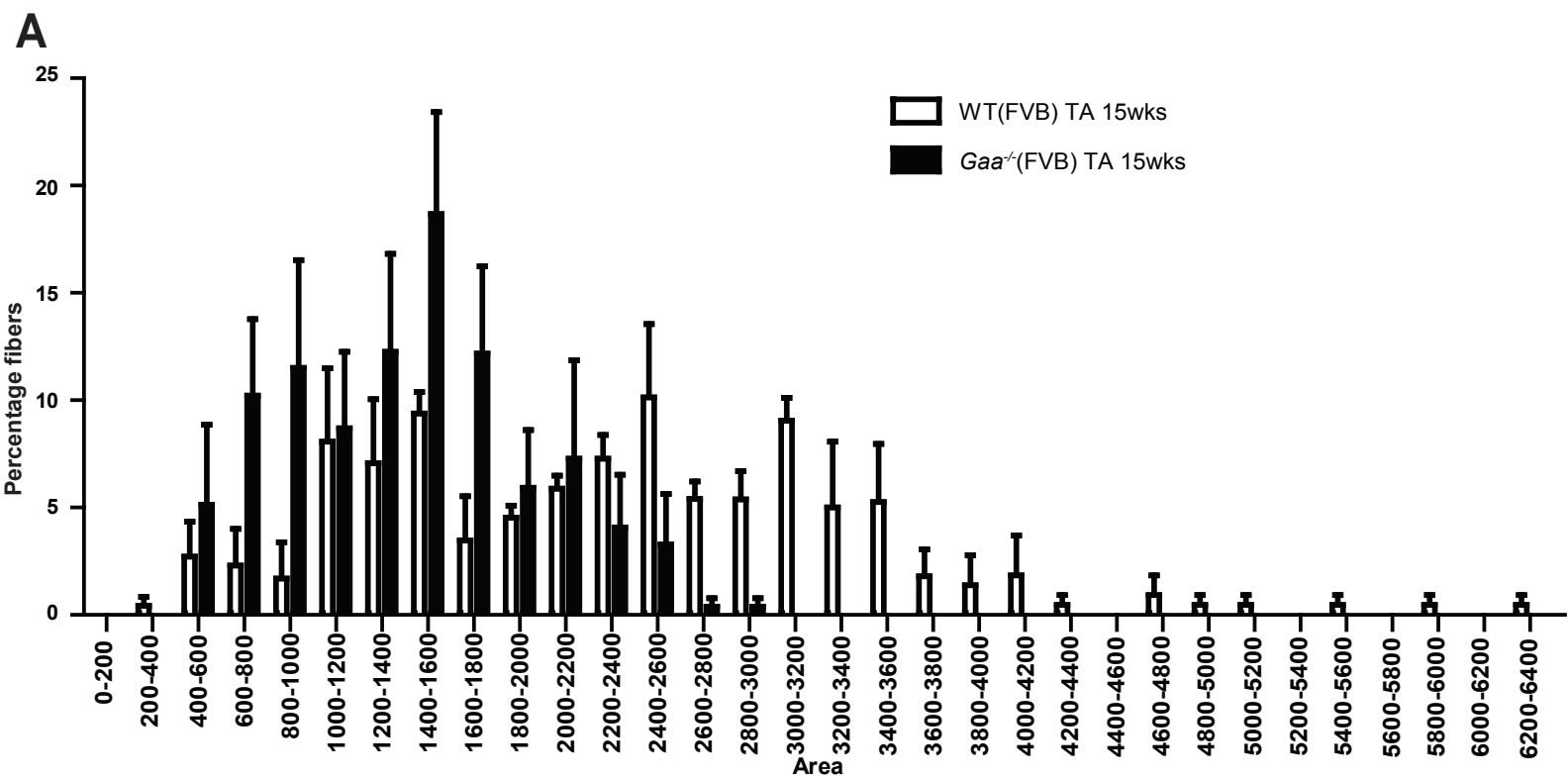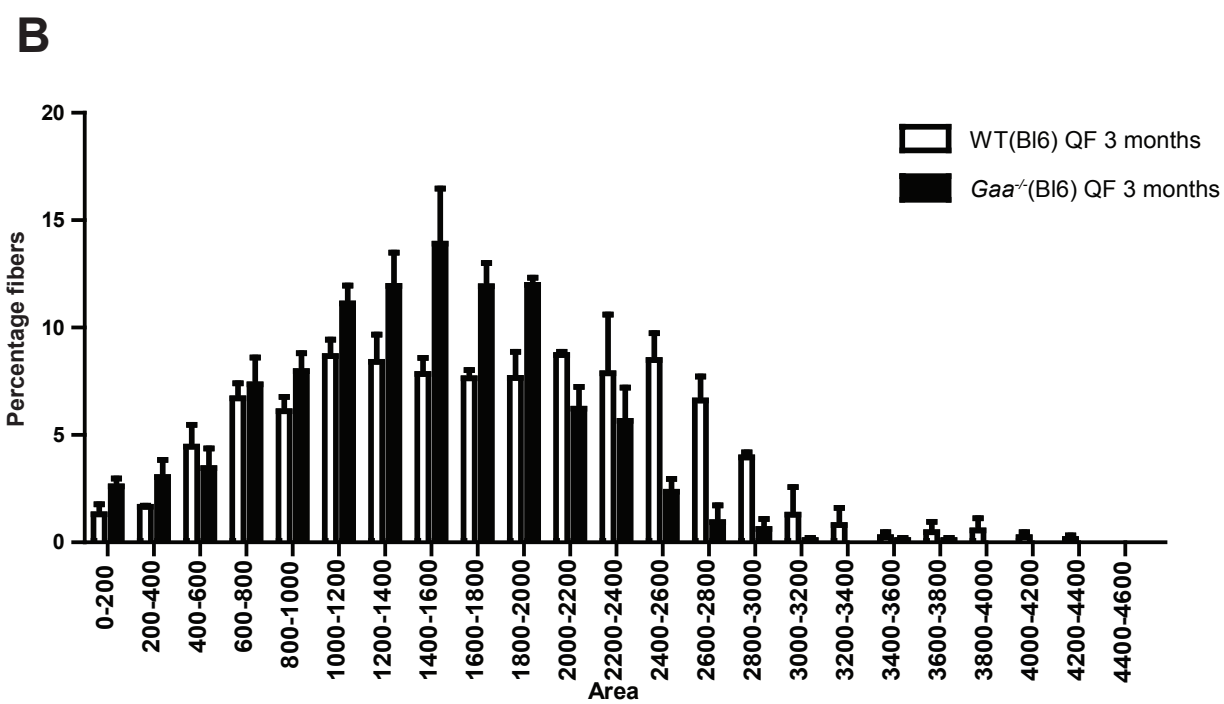

Supplement: Supplementary file 1 — Figure S1. Reduced fiber diameter in GAAKO animals. A. Fiber diameter frequency distribution plot of WT(FVB) and Gaa−/−(FVB) at 15 weeks of age. B. WT(Bl6) and GAAKO(Bl6) at 3 months of age showing reduced fiber diameter in Gaa−/− (Bl6). These data suggest muscle atrophy is observed in GAA-deficient animals on both FVB/N and C57/Bl6 backgrounds. (PDF 122 kb) [file 40478_2018_620_MOESM1_ESM.pdf]

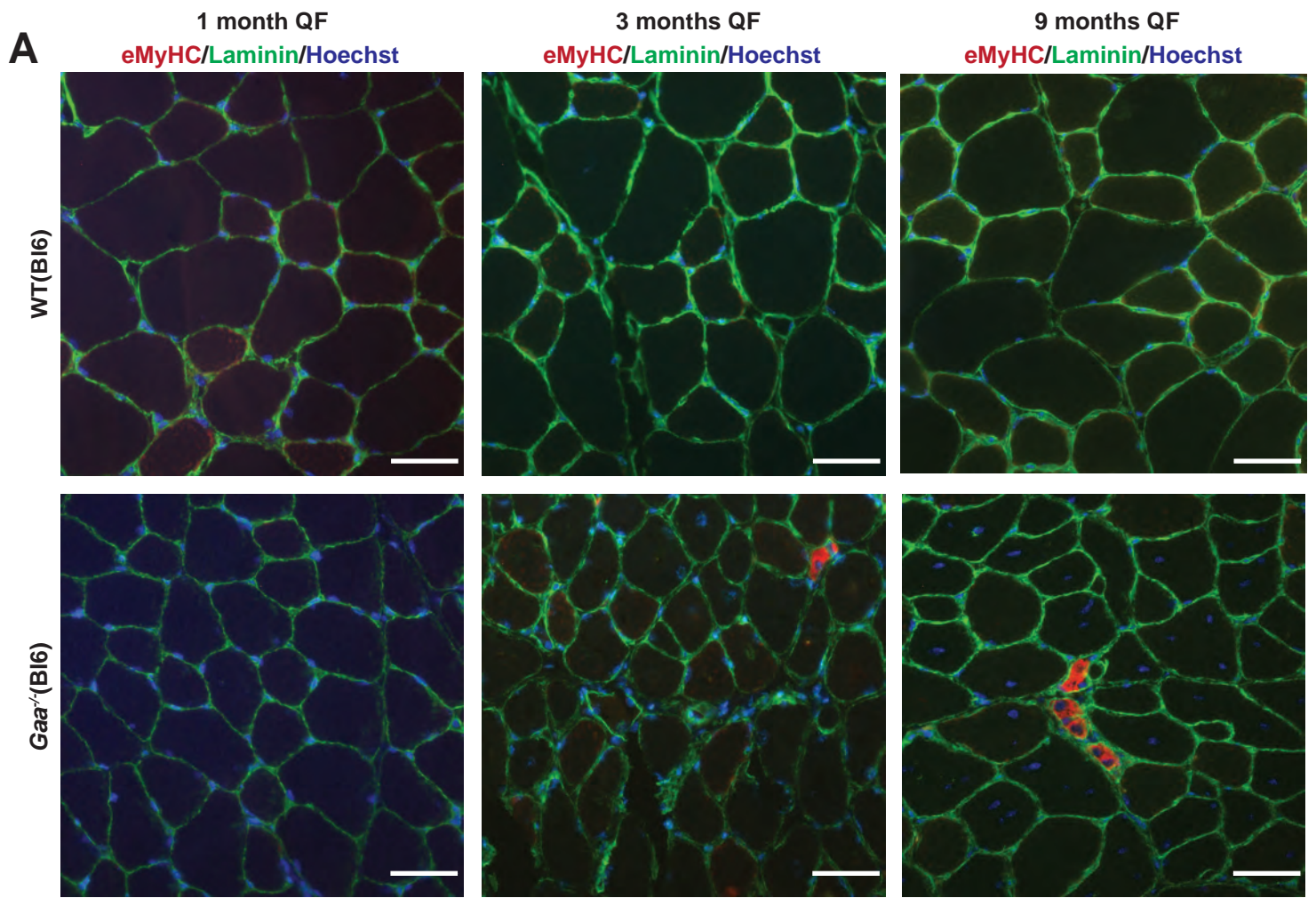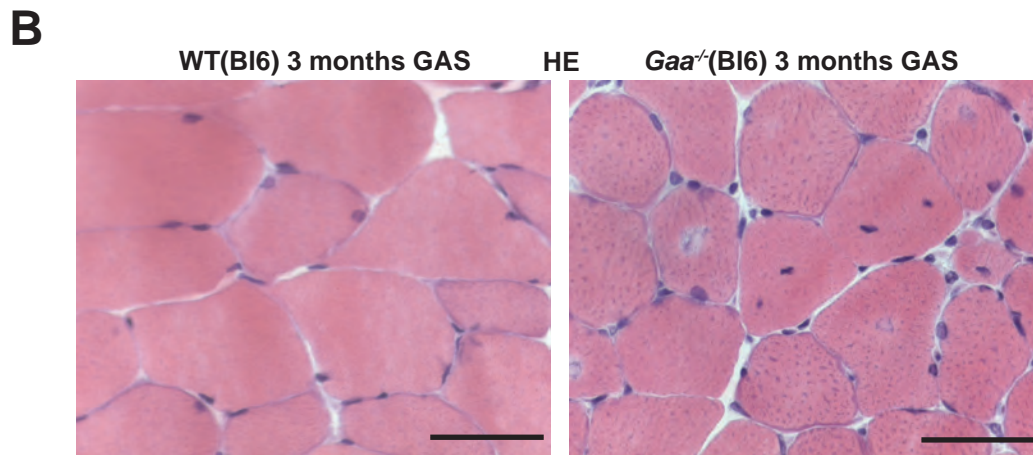

Supplement: Supplementary file 2 — Figure S2. Modest muscle regeneration in Gaa−/−(Bl6) animals. A.eMyHC staining of QF sections from WT(Bl6) and Gaa−/−(Bl6) animals. The figure shows selected areas of eMyHC (red)/laminin(green)/Hoechst(blue) stained QF sections from 4, 12 and 36 week old WT(Bl6) and Gaa−/− (Bl6) animals. eMyHC-positive were rare and very small in Gaa−/− (Bl6) muscle, in line with findings in GAA−/−(FVB) (see Fig. 2A). B. Examples of HE-stained section from GAS muscle from 3 months old WT(Bl6) and Gaa−/−(Bl6) animals. (PDF 320 kb) [file 40478_2018_620_MOESM2_ESM.pdf]

**A**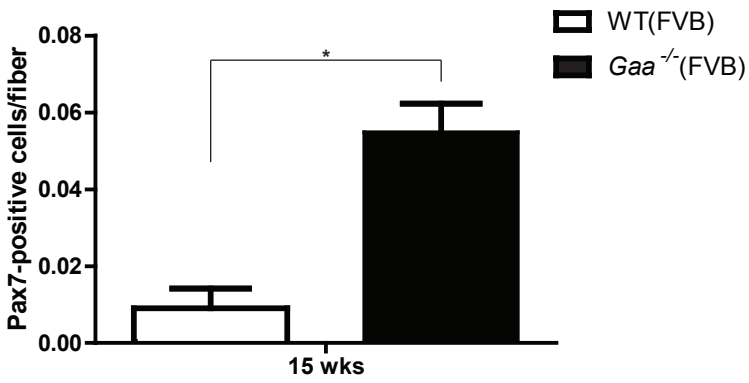**B**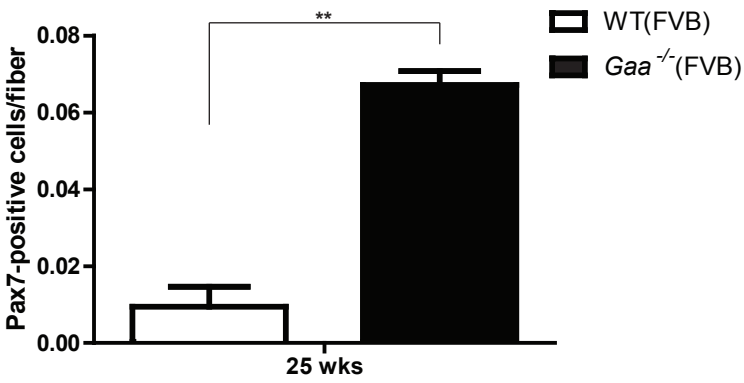

Supplement: Supplementary file 3 — Figure S3. Satellite cell numbers are increased in Gaa−/− TA muscle. The number of Pax7-positive cells in 15 week (A) and 25 week (B) WT and Gaa−/− from Fig. 3 expressed as Pax7-positive cells/myofiber. Data are means ± SD from 2 muscles derived from 2 different animals per genotype per timepoint. *p < 0.05 and **p < 0.01. (PDF 103 kb) [file 40478_2018_620_MOESM3_ESM.pdf]

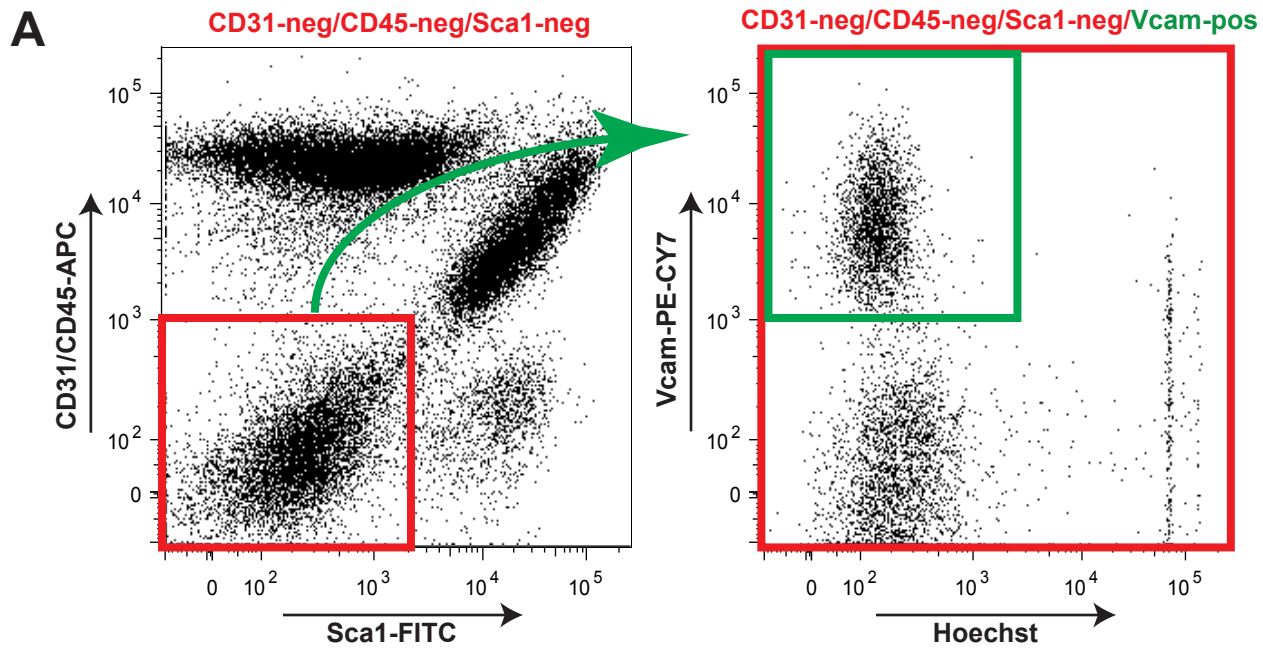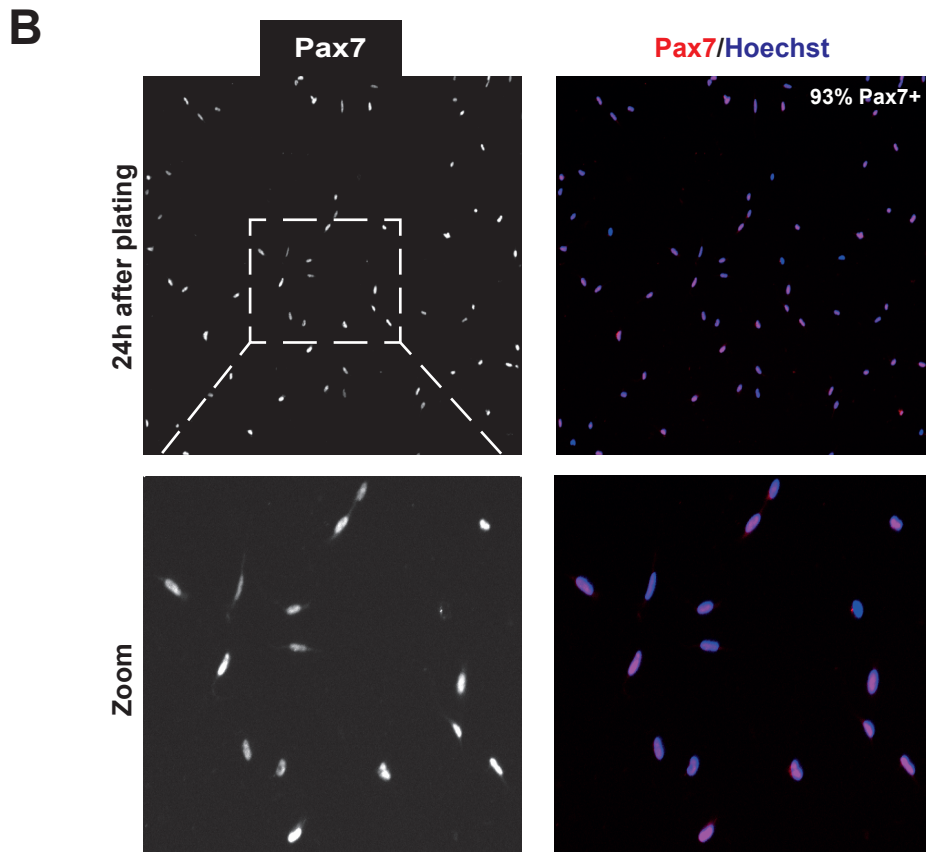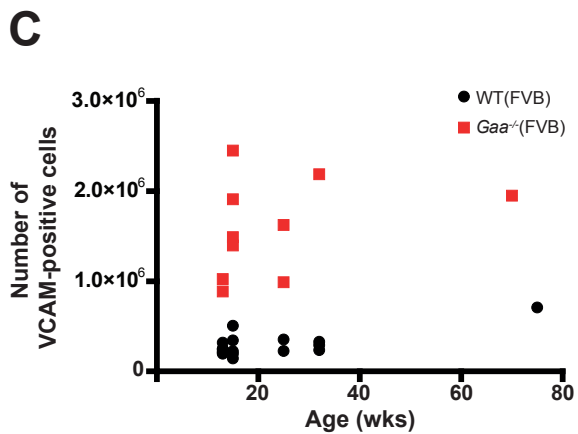

Supplement: Supplementary file 4 — Figure S4. Identification of Pax7-positive satellite cells by flow cytometry. A. Representative dot plots from CD31-APC/CD45-APC/Sca1-FITC/Vcam-PeCY7 stained muscle cell suspensions according to the procedure described previously by Liu et al. [28]. The gating strategy is depicted by the green arrow. The colors of the box/plot outlines correspond with the gated populations. Satellite cells are in the CD45neg/CD31neg/sca1-neg/Vcam-positive gate (green box). B. pax7 (red)/Hoechst(blue) staining of FACS-sorted satellite cells after 24 h culture using the procedure shown in (A). The lower panel shows the zoom of the insert in the upper panel. Counting Pax7 expressing cells indicated that sorting was performed at > 93% purity. C. Quantification of the percentage of Vcam-positive cells by flow cytometry. Data from individual mice are plotted as single dots. (PDF 1056 kb) [file 40478_2018_620_MOESM4_ESM.pdf]

**A**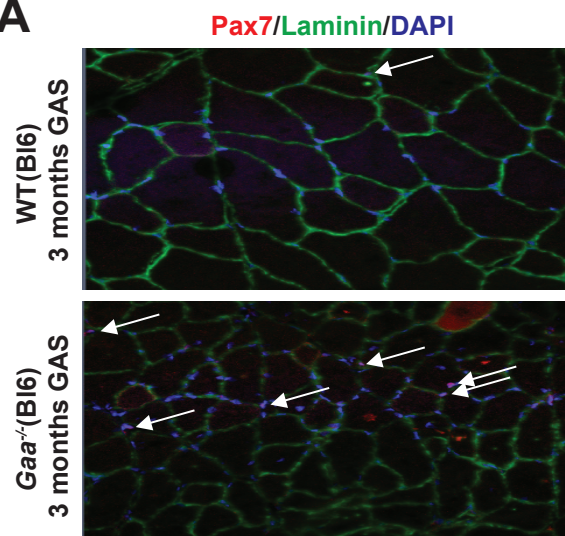**B**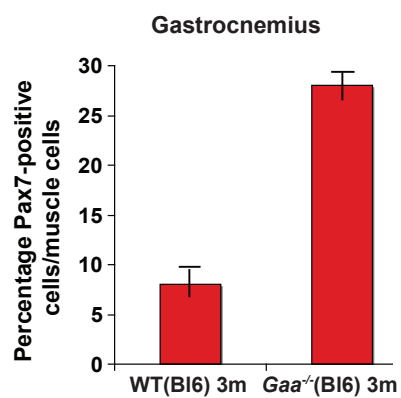**C**Gastrocnemius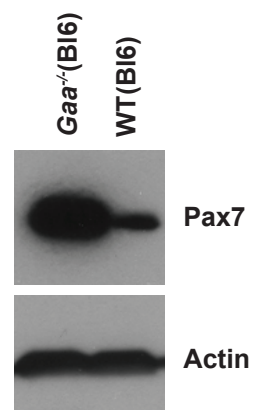

Supplement: Supplementary file 5 — Figure S5. Satellite cell numbers are increased in Gaa−/−(Bl6) muscle. A. Satellite cells were detected in 3 months old WT(Bl6) and Gaa−/−(Bl6) gastrocnemius (GAS) cryosections by immunofluorescent staining of Pax7 (red). Myofibers were visualized using a laminin antibody (green) and nuclei with Hoechst (blue). White arrows point to Pax7-positive satellite cells. B. Quantification of A. The figure depicts the mean percentage of Pax7-positive Satellite cells per field ± SD. C. Western blot analysis of Pax7 expression in GAS muscle from 13 week old WT(Bl6) and Gaa−/−(Bl6) animals. Western blot analysis was performed as previously described [14]. (PDF 952 kb) [file 40478_2018_620_MOESM5_ESM.pdf]

*Gaa*<sup>-/-</sup>(FVB) 15wks TA

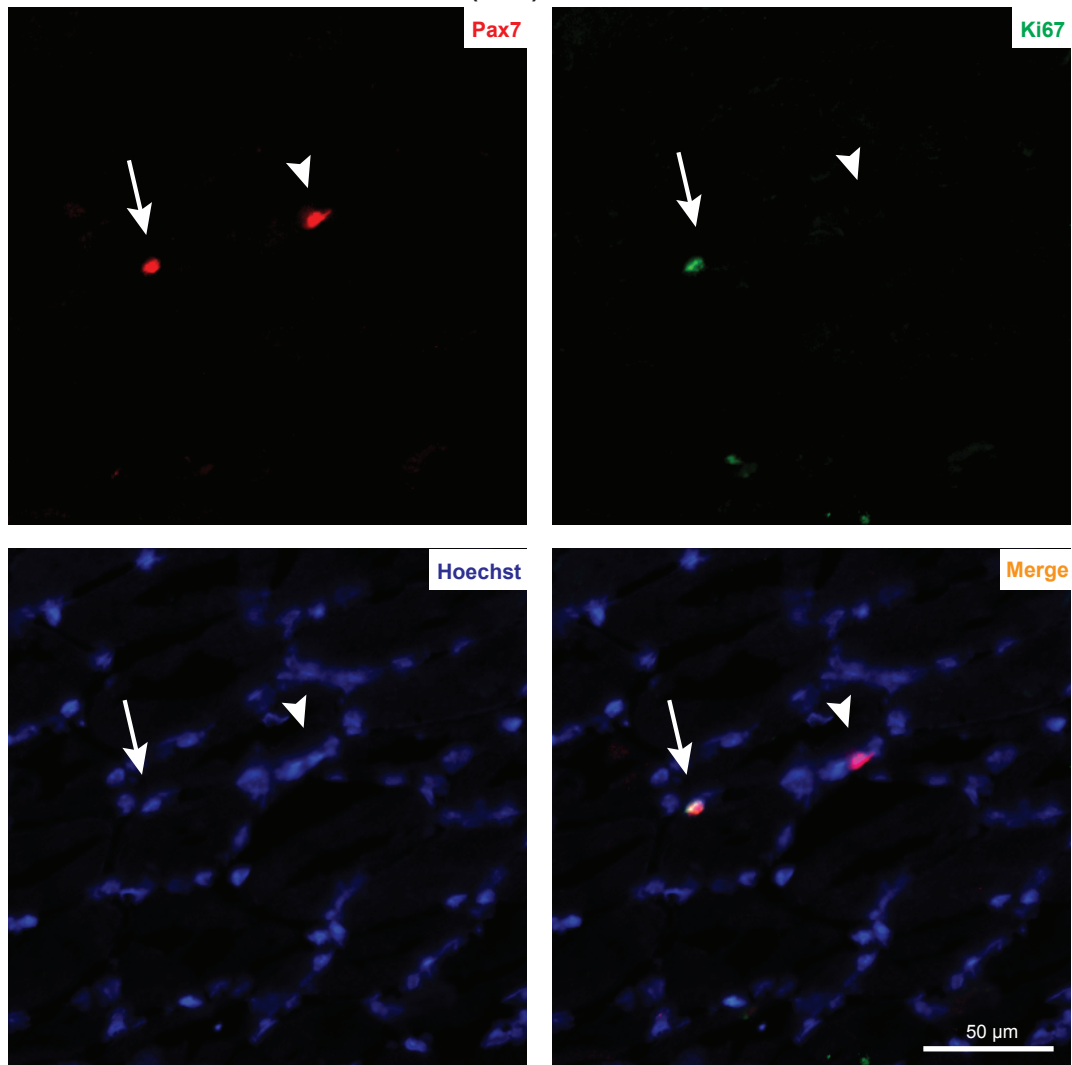

Supplement: Supplementary file 6 — Figure S6. Detection of proliferating satellite cells in GAA-deficient limb muscle. Representative images from TA limb muscle sections co-stained for Pax7 (red) and Ki67 (green) to detect proliferating satellite cells (arrow). Nuclei are visualized with Hoechst (blue). The arrowhead points to a Pax7-positive/Ki67-negative quiescent satellite cell. (PDF 286 kb) [file 40478_2018_620_MOESM6_ESM.pdf]

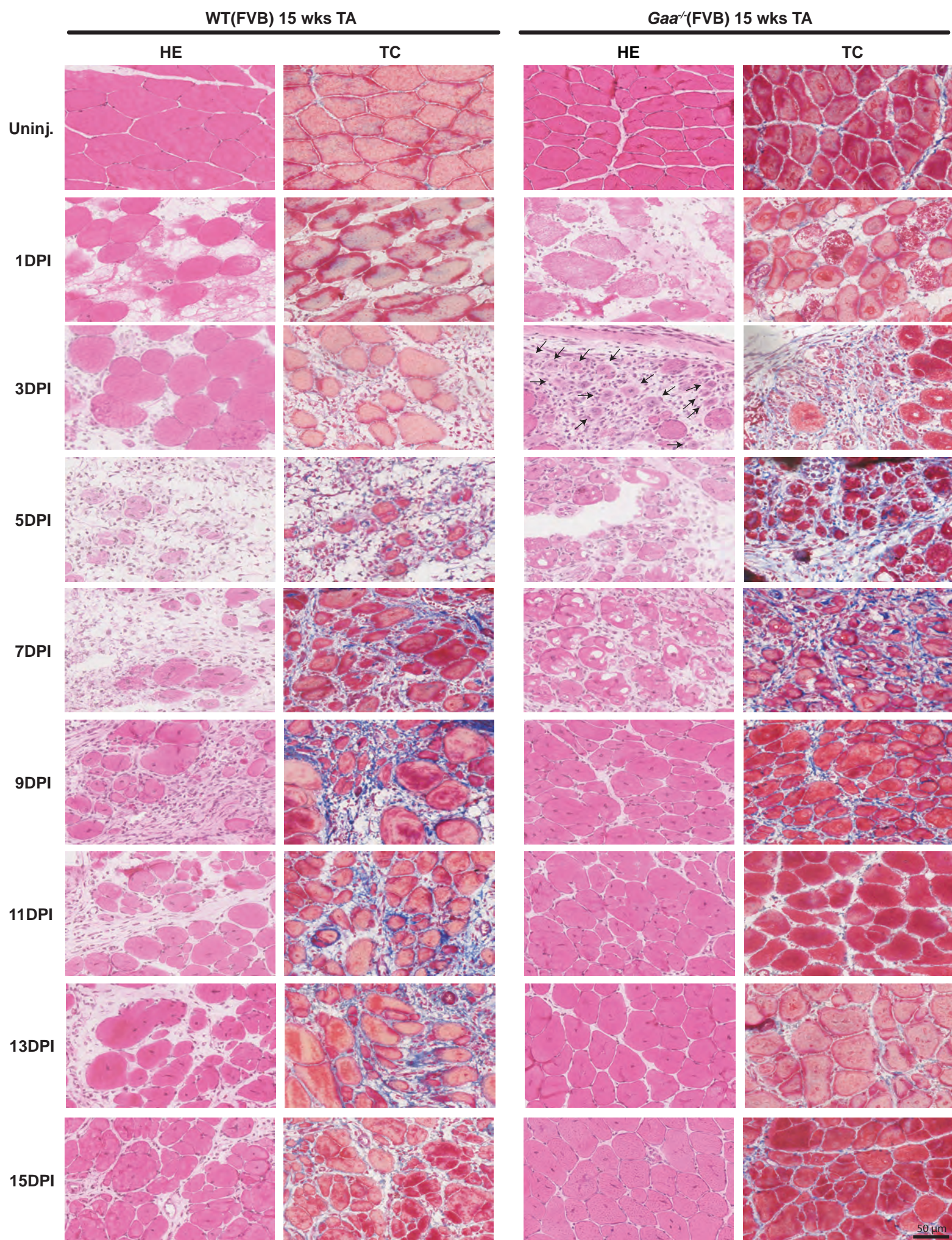

Supplement: Supplementary file 7 — Figure S7. Detailed histological evaluation of regenerating WT and Gaa−/− muscle. The figure depicts HE- and trichrome stained histological sections from 15 week WT(FVB) and Gaa−/− (FVB) TA muscles at multiple time-points during the first 15 days after BaCl2-induced muscle regeneration. Gaa−/− (FVB) muscle regenerates efficiently and completely (left panels). The trichrome stain shows absence of residual fibrotic tissue after completing a regeneration cycle (right panels). As explained in the text WT(FVB) has a regeneration cycle of more than 30 days and was therefore still actively remodelling at 15 DPI. Arrows point to small de novo myofibers detected as early as 3 DPI in regenerating Gaa−/−(FVB) muscle. (PDF 569 kb) [file 40478_2018_620_MOESM7_ESM.pdf]

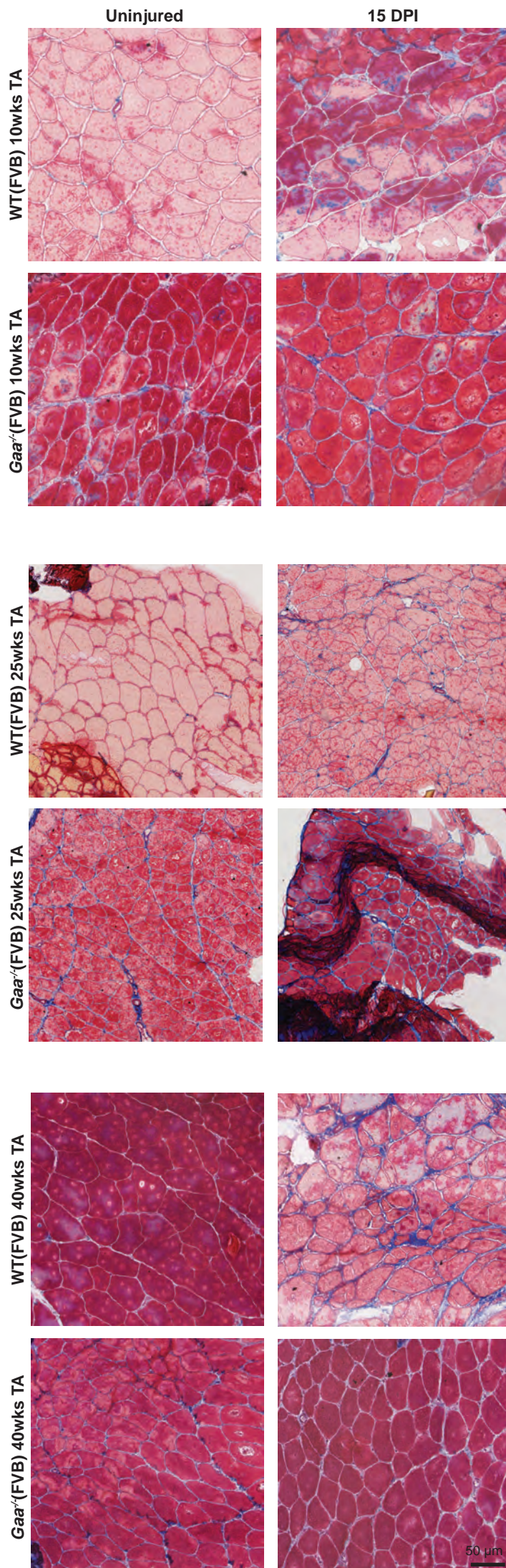

Supplement: Supplementary file 8 — Figure S8. Gaa−/− muscle regenerates completely without tissue remodeling Depicted are images from trichrome staining of TA muscle of WT(FVB) and Gaa−/−(FVB) at 10, 25 and 40 weeks before and 15 days after BaCl2 injury. (PDF 348 kb) [file 40478_2018_620_MOESM8_ESM.pdf]

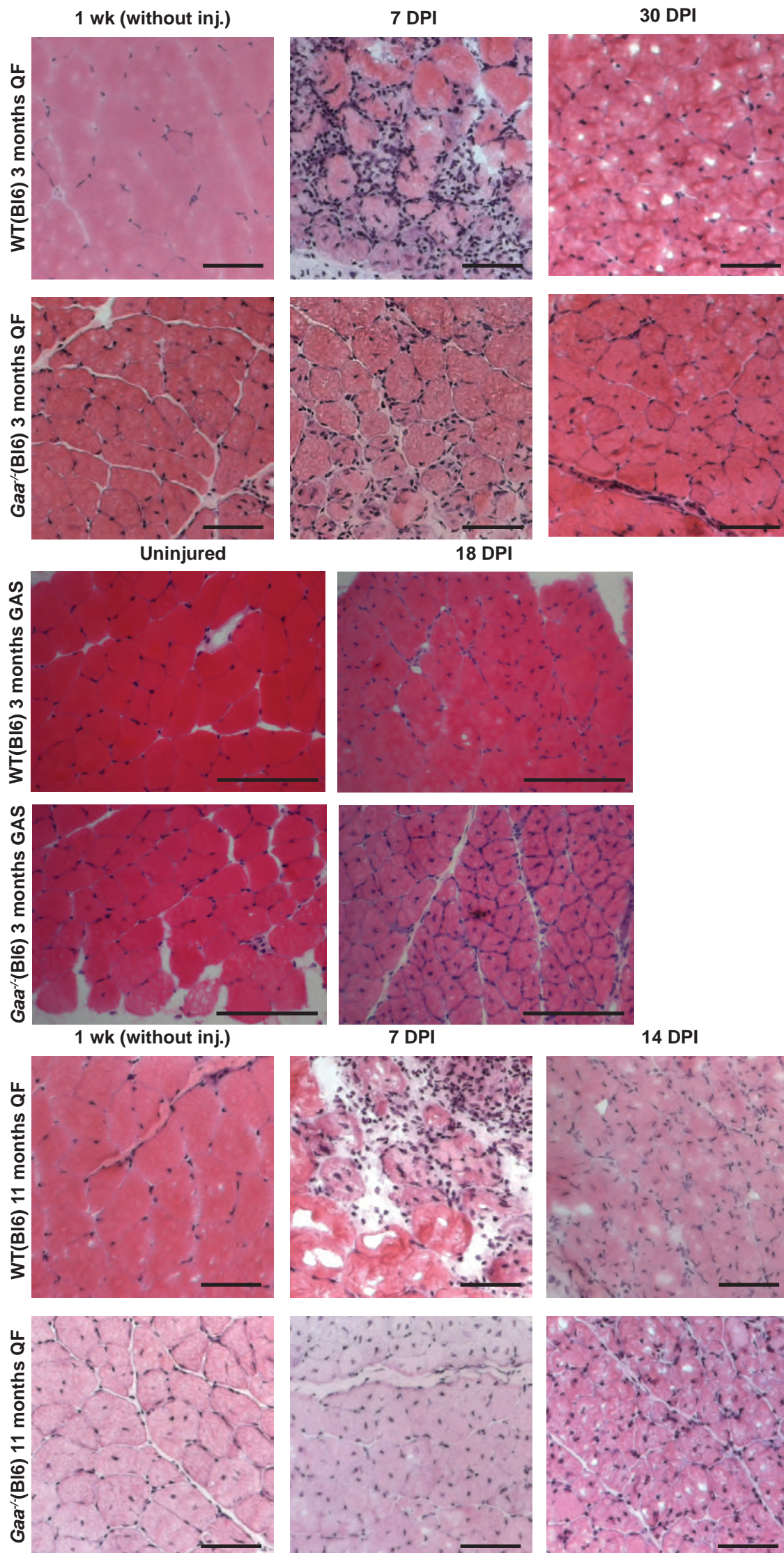

Supplement: Supplementary file 9 — Figure S9. Efficient regeneration of GAAKO-muscle after cardiotoxin-induced injury in GAA-deficient animals on a C57/Bl6 background. The figure shows histological sections from TA HE-stained sections from 12 and 48 week old WT(Bl6) and Gaa−/− (Bl6) animals at indicated time points after injury uisng cardiotoxin (CTX)-injection. The upper panels show HE-stained sections from QF muscle, while the middle panels show images from regenerating GAS. The lower panels depict regenerating QF from 11 months old WT(Bl6) and Gaa−/− (Bl6) animals. These data demonstrate that the capacity to regenerate after experimental injury is also maintained in GAA-deficient muscle on a C57/Bl6 background. (PDF 386 kb) [file 40478_2018_620_MOESM9_ESM.pdf]
